# Successful endoscopic cyanoacrylate injection therapy for ruptured duodenal varices immediately after balloon‐occluded retrograde transvenous obliteration

**DOI:** 10.1002/jgh3.12683

**Published:** 2021-11-18

**Authors:** Yasuyuki Tamai, Hideaki Tanaka, Masashi Fujimori, Motoh Iwasa, Hayato Nakagawa

**Affiliations:** ^1^ Department of Gastroenterology and Hepatology Mie University Graduate School of Medicine Tsu Mie Japan; ^2^ Department of Radiology Mie University Graduate School of Medicine Tsu Mie Japan

**Keywords:** balloon‐occluded retrograde transvenous obliteration, cyanoacrylate, duodenal varices, rebleeding

## Abstract

We present a rare case of acute duodenal variceal rupture after B‐RTO that was successfully treated with endoscopic CA injection therapy. A 74‐year‐old Japanese woman was transferred to our hospital due to progressive general malaise and hematemesis. Gastroduodenoscopy (GDS) showed duodenal varices without active bleeding in the second portion of duodenum. Balloon‐occluded retrograde transvenous obliteration (B‐RTO) was carried out to prevent duodenal variceal rebleeding. Good pooling of ethanolamine oleate with iopamidol (EOI) was observed in duodenal varices using balloon catheters. However, massive melena was observed immediately after B‐RTO. Emergent GDS revealed a white plug on the treated varix, thus endoscopic cyanoacrylate (CA) injection therapy was performed. We speculated that the injection of EOI increased the pressure in the duodenal varices which resulted in rupture of duodenal varices. B‐RTO was effective therapy to prevent duodenal variceal rebleeding, but postprocedural monitoring is required as illustrated by this case. We suggest that careful monitoring and backup system for endoscopy are required during or after B‐RTO.

## Graphical abstract

We present a rare case of acute duodenal variceal rupture after balloon‐occluded retrograde transvenous obliteration (B‐RTO) that was successfully treated with endoscopic cyanoacrylate injection therapy. B‐RTO was effective therapy to prevent duodenal variceal rebleeding, but postprocedural monitoring is required as illustrated by this case. We suggest that careful monitoring and backup system for endoscopy are required during or after B‐RTO.

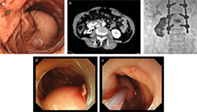

## Introduction

Duodenal varices are one of the complications of portal hypertension secondary to liver cirrhosis. Although the frequency of duodenal varices is lower than that of esophageal and gastric varices,^1^ duodenal varices bleeding is a life‐threatening complication with a mortality of up to 40% for the initial bleeding episode.^2^ Treatment options include endoscopic therapy, interventional radiological therapy, and surgery^1^, ^2^, ^3^ but, there is currently no consensus with regards to optimal treatment for duodenal varices. Hence, we report a case of duodenal variceal rupture immediately after balloon‐occluded retrograde transvenous obliteration (B‐RTO), which was successfully treated with endoscopic cyanoacrylate (CA) injection therapy.

## Case presentation

**Figure 1:**
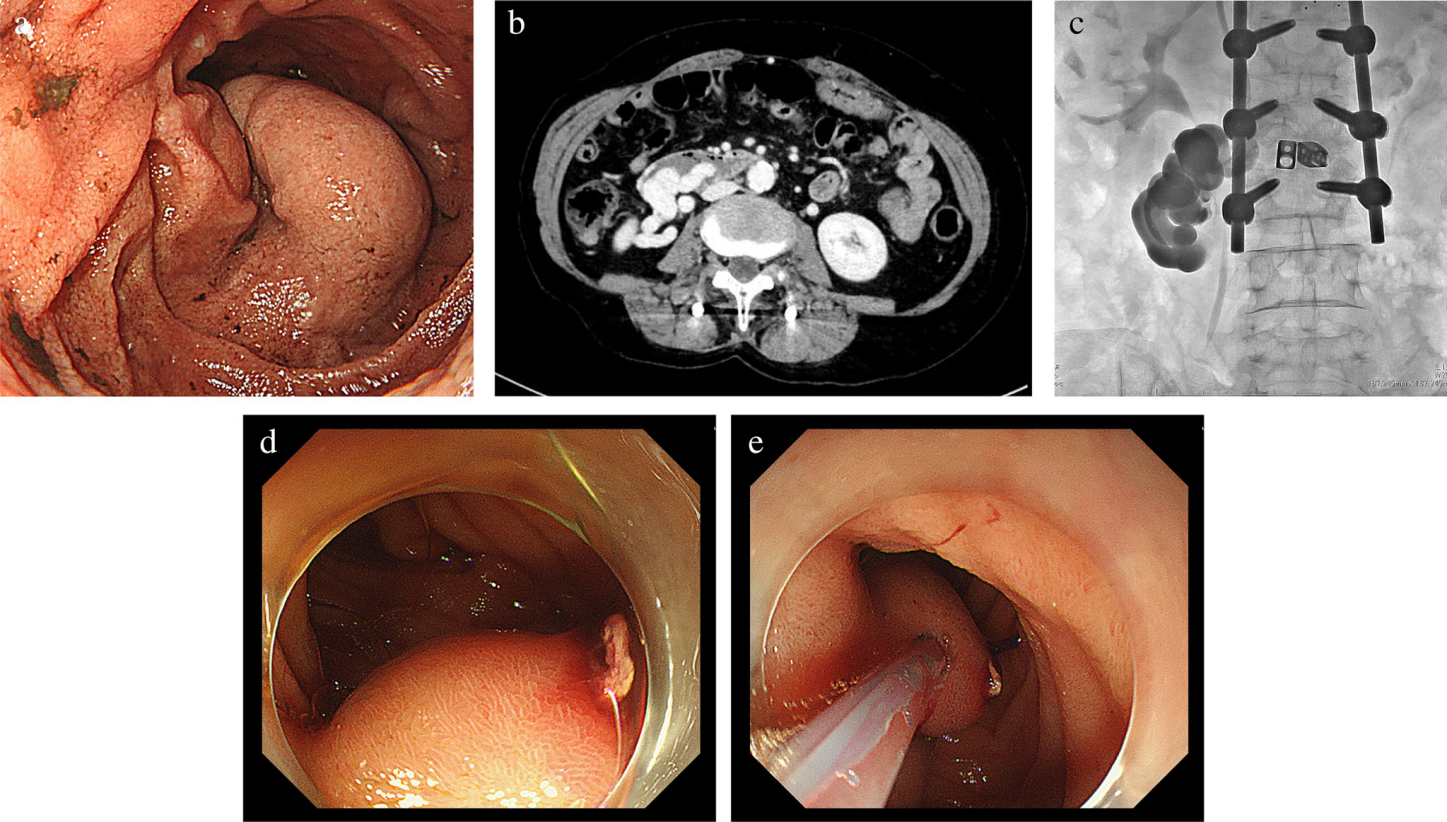
(a) Duodenal varices on admission to a local hospital. (b) Contrast‐enhanced computed tomography (CT) revealed that varices were located in the second portion of the duodenal wall including dilated superior mesenteric veins as a feeding vein and dilated ovarian veins as a drainage vein. (c) B‐RTO showed that good pooling of ethanolamine oleate with iopamidol was observed in duodenal varices using balloon catheters. (d) Emergent gastroduodenoscopy revealed a white plug on the treated varices. (e) A total of 1.5 mL of 66.6% cyanoacrylate was injected carefully into the duodenal varices, and hemostasis was completely achieved.

A 74‐year‐old Japanese woman with progressive general malaise and hematemesis was admitted to a local hospital. She did not have a history of liver disease, but computed tomography (CT) revealed diffuse fatty infiltration of the liver suggestive of cirrhosis. Gastroduodenoscopy (GDS) showed duodenal varices without active bleeding in the second portion of duodenum (Fig. 1a). Laboratory findings at the first local hospital visit indicated moderate anemia. The patient was transferred to our hospital on the following day for further assessments and treatments of duodenal varices.

Laboratory findings on admission to our hospital were as follows: Hemoglobin, 8.5 g/dL; platelets, 118 000/mL; total bilirubin, 0.8 mg/dL; serum albumin, 2.9 g/dL; and prothrombin time, 66.8%. Encephalopathy and ascites were not observed. Liver function was evaluated as grade B according to the Child–Pugh classification. She had no history of alcohol drinking and was negative for hepatitis B surface antigen, as well as anti‐hepatitis C antibody. Contrast‐enhanced CT revealed that varices were located in the second portion of the duodenal wall including dilated superior mesenteric veins as a feeding vein and dilated ovarian veins as a drainage vein. (Fig. 1b).

On the following day, elective B‐RTO was carried out to prevent duodenal varices rebleeding. Retrograde venography via the microcatheter under balloon occlusion was successfully performed to visualize the duodenal varices. Good pooling of ethanolamine oleate with iopamidol (EOI) in duodenal varices was observed using balloon catheters (Fig. 1c). There were no complications during B‐RTO. However, when she returned to her room after B‐RTO, massive melena was observed. Her blood pressure was 71/40 mmHg and pulse was 113/min. Patient was initially managed with fluid resuscitation and organized an emergent GDS. Emergent GDS revealed a white plug on the treated varix (Fig. 1d), thus we performed endoscopic CA injection therapy for the lesion. A total of 1.5 mL of 66.6% CA was injected carefully into the duodenal varices resulted in achieved complete hemostasis (Fig. 1e). In the present case, the procedure was successfully completed without any complications. Subsequently, she was given 6 units of red blood cells, 6 units of fresh frozen plasma, and 20 units of platelets.

During the observation closely for 6 days after treatment, she had no further episodes of melena, maintained hemodynamic state, and remained her hemoglobin consistently. Contrast‐enhanced CT revealed complete thrombosis of duodenal varices with CA precipitation. She was finally discharged from our hospital.

## Discussion

There are no randomized controlled trials (RCT) relating to duodenal varices. Endoscopic CA injection therapy has been reported to achieve hemostasis for duodenal variceal bleeding.^4^, ^5^ However, some studies suggested that endoscopic sclerotherapy may be less effective in duodenal varices management.^1^ Therapy with CA has significant adverse events, including glue embolism and uncontrollable rebleeding.^6^ With regard to gastric varices, B‐RTO is more effective than endoscopic CA injection therapy in order to prevent rebleeding, with similar frequencies of complications in a RCT.^7^ B‐RTO or transjugular intrahepatic portosystemic shunt (TIPS) have been used as a first‐line therapy as recommended by the American Association for the Study of Liver Disease.^8^

B‐RTO have been demonstrated the clinical efficacy and safety. However, complications of B‐RTO may occur, such as rebleeding, pulmonary edema, disseminated intravascular coagulation, portal thrombosis, renal dysfunction, and allergic reaction.^9^ Indeed, we experienced a case of duodenal variceal rebleeding immediately after B‐RTO. We set up a backup endoscopy, and successfully achieved complete hemostasis by endoscopic CA injection therapy. While endoscopists continuously pay attention to glue embolism, CA is injected as needed to ensure hemostasis. We suggest that careful monitoring and backup system for endoscopy are required during or after B‐RTO as illustrated by this case.

## References

[1] Norton ID , Andrews JC , Kamath PS . Management of ectopic varices. Hepatology. 1998; 28: 1154–8. 9755256 10.1002/hep.510280434

[2] Khouqeer F , Morrow C , Jordan P . Duodenal varices as a cause of massive upper gastrointestinal bleeding. Surgery. 1987; 102: 548–52. 3498234

[3] Kakizaki S , Toyoda M , Ichikawa T *et al*. Clinical characteristics and treatment for patients presenting with bleeding duodenal varices. Dig. Endosc. 2010; 22: 275–81. 21175479 10.1111/j.1443-1661.2010.01007.x

[4] Soga K , Tomikashi K , Fukumoto K *et al*. Successful endoscopic hemostasis for ruptured duodenal varices after balloon‐occluded retrograde transvenous obliteration. Dig. Endosc. 2010; 22: 329–33. 21175490 10.1111/j.1443-1661.2010.01023.x

[5] Malik A , Junglee N , Khan A , Sutton J , Gasem J , Ahmed W . Duodenal varices successfully treated with cyanoacrylate injection therapy. BMJ Case Rep. 2011; 2011: bcr0220113913. 10.1136/bcr.02.2011.3913 PMC3109690 22694885

[6] Seewald S , Ang TL , Imazu H *et al*. A standardized injection technique and regimen ensures success and safety of *N*‐butyl‐2‐cyanoacrylate injection for the treatment of gastric fundal varices (with videos). Gastrointest. Endosc. 2008; 68: 447–54. 18760173 10.1016/j.gie.2008.02.050

[7] Luo X , Xiang T , Wu J *et al*. Endoscopic cyanoacrylate injection vs BRTO for prevention of gastric variceal bleeding: a randomized controlled trial. Hepatology. 2021; 74: 2074–84. 33445218 10.1002/hep.31718

[8] Garcia‐Tsao G , Abraldes JG , Berzigotti A , Bosch J . Portal hypertensive bleeding in cirrhosis: risk stratification, diagnosis, and management: 2016 practice guidance by the American Association for the Study of Liver Diseases. Hepatology. 2017; 65: 310–35. 27786365 10.1002/hep.28906

[9] Hirota S , Kobayashi K , Kako Y , Takaki H , Yamakado K . Balloon‐occluded retrograde transvenous obliteration of varices: focusing on the portal hemodynamics and the recent techniques. Hepatol. Int. 2018; 12: 102–11. 28875380 10.1007/s12072-017-9813-2

